# Specificity of affective dynamics of bipolar and major depressive disorder

**DOI:** 10.1002/brb3.3134

**Published:** 2023-08-13

**Authors:** Emma K. Stapp, Vadim Zipunnikov, Andrew Leroux, Lihong Cui, Mathilde M. Husky, Debangan Dey, Kathleen R. Merikangas

**Affiliations:** ^1^ Genetic Epidemiology Research Branch National Institute of Mental Health Bethesda Maryland USA; ^2^ Department of Epidemiology, Milken Institute School of Public Health George Washington University Washington, D.C. USA; ^3^ Department of Biostatistics Johns Hopkins Bloomberg School of Public Health Baltimore Maryland USA; ^4^ Department of Biostatistics and Informatics University of Colorado School of Public Health Aurora Colorado USA; ^5^ Bordeaux Population Health Research Center University of Bordeaux Bordeaux France

**Keywords:** affective symptoms, bipolar disorder, depressive disorder, ecological momentary assessment, emotions, major, structural equation modeling

## Abstract

**Objective:**

Here, we examine whether the dynamics of the four dimensions of the circumplex model of affect assessed by ecological momentary assessment (EMA) differ among those with bipolar disorder (BD) and major depressive disorder (MDD).

**Methods:**

Participants aged 11–85 years (*n* = 362) reported momentary sad, anxious, active, and energetic dimensional states four times per day for 2 weeks. Individuals with lifetime mood disorder subtypes of bipolar‐I, bipolar‐II, and MDD derived from a semistructured clinical interview were compared to each other and to controls without a lifetime history of psychiatric disorders. Random effects from individual means, inertias, innovation (residual) variances, and cross‐lags across the four affective dimensions simultaneously were derived from multivariate dynamic structural equation models.

**Results:**

All mood disorder subtypes were associated with higher levels of sad and anxious mood and lower energy than controls. Those with bipolar‐I had lower average activation, and lower energy that was independent of activation, compared to MDD or controls. However, increases in activation were more likely to perpetuate in those with bipolar‐I. Bipolar‐II was characterized by higher lability of sad and anxious mood compared to bipolar‐I and controls but not MDD. Compared to BD and controls, those with MDD exhibited cross‐augmentation of sadness and anxiety, and sadness blunted energy.

**Conclusion:**

Bipolar‐I is more strongly characterized by activation and energy than sad and anxious mood. This distinction has potential implications for both specificity of intervention targets and differential pathways underlying these dynamic affective systems. Confirmation of the longer term stability and generalizability of these findings in future studies is necessary.

## Graphical abstract

We investigated multivariate emotional dynamics across the full circumplex of affect in a euthymic, community sample of persons with and without mood disorders. We used dynamic structural equation modeling to differentiate patterns of affective fluctuation between mood disorder subtypes as reported via ecological momentary assessment. Findings underscore a primacy of energy/activation rather than mood/valence particularly for bipolar I, with substantive differences between bipolar subtypes and between bipolar and major depressive disorders.

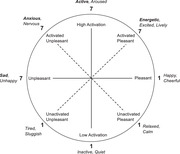

## 1 INTRODUCTION

Hallmark features of bipolar disorder (BD) involve fluctuation of mood, cognition, and activity, whereas major depressive disorder (MDD) is characterized by episodes of lower mood, cognition, and activity (American Psychiatric Association, 2000, 2013,2000, 2013). This fluctuation not only defines acute episodes, but also may influence interepisode function (Cochran et al., 2016; Henry et al., 2008; Prisciandaro et al., 2019; Sperry et al., 2020; Taylor et al., 2021; Wichers et al., 2010). This highlights the importance of better understanding affective fluctuation in mood disorder subtypes, with its potential for illuminating underlying differences between disorders and targets for intervention.

There are two major challenges to measuring affective fluctuation. The first is that it may not be well‐captured by reports that rely on retrospective assessments across a long reporting window—especially in the context of active fluctuation or occasional problems with insight. And current affective states can influence participants’ appraisal of their trait affect, underscoring the value of repeat measures (Brose et al., 2013). There has been increasing use of ecological momentary assessment (EMA), which involves many repeated measures within and between days that facilitate collection of dynamics of mood states in real time (Johns et al., 2019; Lamers et al., 2018; Merikangas et al., 2019). By design, EMA is less vulnerable to recall bias and has better ecological validity than retrospective assessments; it is also particularly well‐suited to capturing implicit intraindividual processes and real‐time dynamics (Burchert et al., 2021; Myin‐Germeys et al., 2009; Schneider et al., 2020; Trull & Ebner‐Priemer, 2020). The second challenge is that affect is a complex, multifaceted construct. A circumplex model of affect posits that much of emotional experience can be captured by dimensions of pleasant to unpleasant valence and high to low activation, which are orthogonal, and their product (Feldman Barrett & Russell, 1998; Larsen & Diener, 1992; Posner et al., 2005; Russell, 1980; Yik et al., 1999). Larsen and Diener's (1992) circumplex involves a symmetric and fully dimensional approach, labeling subjective feeling tone (valence) and activation (arousal) and their combinations. Using four bipolar scores to represent the four major dimensions thereby represents the full circumplex structure of affective states.

Despite the relevance of theory‐informed design of EMA studies of affective dynamics (Hall et al., 2021) and comprehensiveness of the full circumplex, prior studies have tended to rely on either one or two indicators of mood or scales of positive and negative affect composed of a wide variety of indicators. This neglects the multifaceted nature of affect, and may obscure the driving components and reduce comparability across studies with different composite indicators. Evidence from such studies using summary scales or broad meta‐analytic categories suggests associations between MDD with higher average levels, range (variability), and lability (temporal instability) of negative affect (Aan Het Rot et al., 2012; Houben et al., 2015; Scott et al., 2020; Thompson et al., 2012) and lower positive affect (Aan Het Rot et al., 2012; Thompson et al., 2012). These same summary approaches link BD or (hypo)mania with variability of positive emotions and variability and instability of negative affect (Gruber et al., 2013; Houben et al., 2015; Knowles et al., 2007; Sperry & Kwapil, 2022). These prior findings on euthymic affective dynamics in BD are more mixed than those in depression research, which may be due in part to the need to disambiguate individual components of the circumplex. Moreover, earlier reports, including our own work (Lamers et al., 2018), modeled inertia, variability, and instability separately and without cross‐lags. That prevented concurrent examination of interrelationships of affective states themselves and their forms of variability.

Substantial evidence from community and clinical samples has documented distinctions in the familiality and genetics, onset and course, consequences, patterns of comorbidity, and treatment response between the BD subtypes of bipolar disorder type I (BPI) and bipolar disorder type II (BPII) as well as between BD and MDD (Mcintyre et al., 2022; Merikangas et al., 2014; Song et al., 2018; Tondo et al., 2022). Our earlier work distinguished different patterns in people with a history of BPI, BPII, MDD, and controls, including greater changes in sad mood and energy in response to changes in motor activity among persons with BPI (Merikangas et al., 2019). However, that work considered sad mood and energy separately without including their dynamic interrelationships.

We therefore employed dynamic structural equation modeling (DSEM) (Asparouhov et al., 2017) to examine the full emotional circumplex and the interrelationships in the underlying dimensions in persons with each of the major mood disorder subtypes. This approach combines time‐series, multilevel, and structural equation modeling to allow for individual differences in parameters that define an individual's time‐series process, thereby providing myriad possibilities without the strict assumptions of prior approaches to modeling EMA data. In a euthymic community‐based sample enriched for mood disorders, our specific aims were to (1) characterize unconditional real‐time affective dynamics stratified by mood disorder subtype among all four dimensions from the mood circumplex and (2) identify significant differences in those affective dynamics conditional on mood disorder subtypes. Based on phenotypic and dynamic differences across mood disorders described above, we hypothesized that there would be differences in activation between BD and MDD and in valence between BPI and BPII, as well as unique patterns of emotional cross‐reactivity among mood disorder subtypes.

## 1 METHODS

### 1.1 Sample

The sample for the present study is a subset (*N* = 362) of participants from the National Institute of Mental Health (NIMH) Family Study of Affective Spectrum Disorders, a community‐based study enriched for probands with mood disorders. Specifically, the current sample is composed of participants with data from both the directly ascertained diagnostic interviews and the first wave of ambulatory monitoring (EMA). The broader study began enrollment in 2004 and is still open; however, data for this analysis were collected through December 7, 2021. Recruitment and procedures are described in detail elsewhere (Merikangas et al., 2014, 2019,2014, 2019). Briefly, probands were recruited from a community screening of the greater Washington, D.C. area, local health newsletters and announcements, the National Institutes of Health Clinical Center general volunteer core, and the NIMH Mood and Anxiety Program. Inclusion criteria were ability to speak English, availability to themselves participate, and consent to contact at least two living first‐degree relatives. The study was approved by the Combined Neuroscience Institutional Review Board at the National Institutes of Health. Adult participants provided written informed consent, and minors provided assent in addition to guardian consent.

### 1.2 Measures and procedure

#### 1.2.1 Psychiatric diagnostic interview

The NIMH Family Study Diagnostic Interview for Affective Spectrum Disorders ascertains diagnostic criteria as well as subthreshold phenomenology related to current and lifetime disorders from the Diagnostic and Statistical Manual of Mental Disorders (DSM‐IV) (American Psychiatric Association, 2000). The interview was developed based on earlier diagnostic interviews for genetic epidemiologic studies such as the Schedule for Affective Disorders and Schizophrenia and the Diagnostic Interview for Genetic Studies and does not adhere to skip‐outs based on frequency or duration at the probe level. Experienced clinicians conducted direct interviews of probands and direct, blinded interviews of relatives. There is a child version and an adult version. Interrater reliability of all diagnostic categories was high, with intraclass correlations of .87 and above across all diagnostic categories. Best estimate DSM‐IV diagnoses were based on all available information, including direct interviews, family history, and consensus ratings from experienced clinicians. The present study includes individuals with lifetime BPI, BPII, or MDD, as well as classical “controls” without any lifetime major psychiatric disorders or syndromes.

#### 1.2.2 Ecological momentary assessment

The participants were not given these procedures if experiencing an episode; safety of the participant was paramount, given the observational nature of the study. For 14 consecutive days, participants used a preprogramed mobile device that administered brief electronic interviews four times per day at fixed intervals. The devices were either the Palm Tungsten E2 personal digital assistant or Samsung Galaxy Player 4.2 Android. The four daily assessments occurred approximately every four waking hours, with schedules adapted to accommodate participants’ typical sleep/wake cycles and a small amount of random variation within minutes around the set times. For example, a participant could select a schedule with assessments at approximately 7:00 a.m., 11:00 a.m., 3:00 p.m., and 7:00 p.m. Study staff trained participants how to use the devices and fill out the EMA questionnaires. Comprehensive reviews have suggested that there is not a single exemplary design for EMA studies of mood disorders; rather, important information is obtained from both interval‐ and signal‐contingent recording of dynamic constructs (Hall et al., 2021).

**FIGURE 1:**
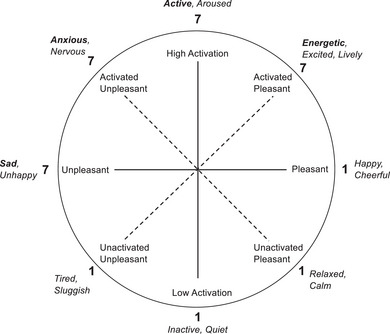
Circumplex model of affect overlaid with analytic variables showing dimension and naming convention. Adapted from Larsen and Diener (1992), which was adapted from Russell (1980) and Watson and Tellegen (1985). Dimensions within the circumplex are as described by Larsen and Diener, and italicized words outside the circumplex are the individual emotions presented in our ecological momentary assessment (EMA) questionnaire. Words additionally bolded reflect our naming conventions for the variables/dimensions, with the scores from the Likert‐type scale.

The set of EMA questions regarding momentary affect used in this analysis were the same across both devices, representing the four major emotional dimensions with eight corresponding octants of the mood circumplex (Larsen & Diener, 1992; Figure 1). Participants rated from 1 to 7 on a Likert‐type scale to indicate how they were feeling at the moment: happy versus **sad** (*Pleasant* to *Unpleasant*), relaxed versus **anxious** (*Unactivated Pleasant* to *Activated Unpleasant*), inactive versus **active** (*Low Activation* to *High Activation*), and tired versus **energetic** (*Unactivated Unpleasant* to *Activated Pleasant*). Results are discussed in terms of more or less of the bolded variable name. Each of the momentary mood variables were latent person‐mean centered in Mplus, which rescales values so that the value at each assessment represents deviation from the person's mean. This facilitates understanding of within‐person processes—for example, whether a person's fluctuating sadness, relative to their typical sadness, influences their anxiousness at the next timepoint, irrespective of its relativity to the group's average sadness. This process does not transform the variance. Latent person‐mean centering is similar to observed person‐mean centering where each person's sample mean is subtracted from the raw values across timepoints; however, latent variables for the between‐level components are introduced to account for measurement error such that the mean estimate is as accurate as possible, eliminating Nickell's bias and Ludtke's bias and accommodating missing data (Asparouhov et al., 2017).

#### 1.2.3 Covariates

Age and sex (female, male) were included as covariates in predictor modeling.

### 1.3 Statistical analyses

#### 1.3.1 Bayesian approach

DSEM is a framework implemented in Mplus to analyze intensive longitudinal data, incorporating time‐series modeling to account for dynamics or lagged associations within the data from one individual over time, multilevel modeling to account for multiple persons and allowing for between‐person differences, and structural equation modeling to facilitate path and factor analyses of those between‐person differences (Asparouhov et al., 2017; Hamaker & Muthen, 2021). The DSEM model is estimated with Bayesian methods, based on the Markov chain Monte Carlo algorithm via Gibbs sampler. Diffuse priors were implemented by default in Mplus, which was an acceptable approach for our models because of adequate sample size to inform the posteriors via likelihood (Schultzberg & Muthén, 2017). The analyses in this study made use of at least 5000 iterations, two chains, with a thinning of 10 (i.e., one in 10 iterations was saved). Additionally, we conducted a sensitivity analysis with at least 10,000 iterations of each model; these produced nearly identical results and we present them side‐by‐side with our main results in Tables S1 and S2. At each iteration of the Markov chain Monte Carlo algorithm, missing data are sampled from their conditional posterior, which takes into account an individual's neighboring observations and autocorrelation as well as residual variance. DSEM does not use listwise deletion and can accommodate data missing at random. For this study, timepoints were treated as being equally spaced given their representation of approximately four waking hours each. In simulations, more densely spaced time grids could be inserted with reasonable estimation with as much as 80% missing data (Asparouhov et al., 2017), but our approach for the current study involved a naturalistic grid based on wakeful hours, where missing data would only exist for actual missed timepoints to preserve interpretability. Certain data are available on request; they are not publicly available due to privacy.

#### 1.3.2 Model decomposition and interpretation

**FIGURE 2:**
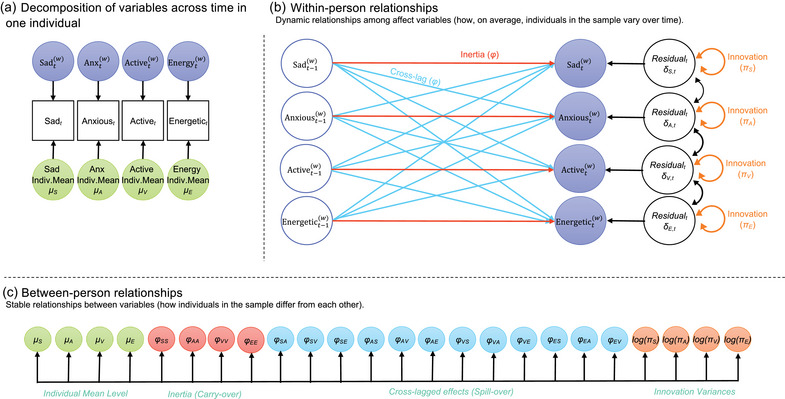
Dynamic structural equation model (DSEM) of affective dynamics. DSEM adapted from Hamaker et al. (2018) demonstrating lag‐1 multilevel vector autoregressive model (VAR(1)) of affect. Panel A shows the decomposition of the intensive longitudinal data into dynamic within‐person (time‐varying) and stable between‐person (time‐invariant) components. Panel B shows the within‐person level model, with relationships between the time‐varying components of these variables. Panel C shows the between‐person‐level model, which includes both the between‐person observed individual means and the random effects from the within‐person‐level model. Innovation variances at the within‐person level are modeled using their log at the between‐person level to ensure they are positive for all individuals.

Figure 2 demonstrates the DSEM model of affective dynamics (Hamaker et al., 2018). This is a lag‐1 multilevel vector autoregressive model (VAR(1)) with random individual means (*μ*), autoregressive and cross‐lagged (*φ*) regression coefficients, and log of the variance of the innovations (log(*π*)) for momentary emotional dimensions (sad, anxious, active, energetic). Random refers to the effects being allowed to vary across individuals. *Individual means* are the within‐person mean level of a given emotional dimension across all timepoints, such as sadness (*μ*_S_), which are modeled at the between‐person level (in essence, group means of individual mean levels). Autocorrelation is the within‐person relationship from one timepoint to the next, via the autoregression parameters, which uses past values to predict the current value. It can also be referred to as *inertia* or carryover, for example, the extent to which an individual's mood state such as sadness carries over from one timepoint to the next (*φ*_SS_). Higher positive inertia suggests it takes longer to return to equilibrium after an increase at the prior timepoint. At the within‐person level, we also measure the way a given indicator of momentary mood is influenced by a different indicator of momentary mood at a prior timepoint; this is referred to as the cross‐regression, representing the relationships across mood states and time, or *cross‐lags*. For example, we measure to what extent prior anxiety spills over into subsequent sadness (*φ*_SA_). *Innovation* variances on the within‐person level include everything that was not measured explicitly but affects the course of the observed variables, such as individual variability of exposures and reactivity to those influences or external factors (Hamaker et al., 2018), and reflect within‐person intensity of change from timepoint to timepoint, or lability. Innovation variances at the within‐person level are modeled at the between‐person level using their log to ensure they are positive for all individuals.

As presented in the results, means are the fixed or averaged effects in the sample or stratum for each of the random effects; variances of those means (shown in Tables S1 and S2) reflect the variability between the participants for each of the random effects. Tables include log innovation variances, with select exponentiated numbers presented here in the text. All means and variances in unconditional stratified models and regression coefficients in predictor modeling include 95% credible intervals (CIs), which can be interpreted as there being a 95% probability that the true (unknown) estimate would lie within the interval, given the evidence provided by the observed data, or, stated another way, given the observed data the effect has 95% probability of falling within the interval. Credible intervals that do not contain zero provide evidence that the mean differs from zero; however, when group means do not differ from zero it does not imply that the parameter is unimportant, because there may be meaningful individual differences.

Because all forms of correlation are measured simultaneously in the VAR(1) model, the effect estimates produced account for all cross‐lags, inertias, innovations, and group means of individual mean levels of all four emotional dimensions and their covariances at once. Unconditional models present all forms of emotional variability and cross‐reactivity stratified by BPI, BPII, BD (a composite variable of BPI and BPII), MDD, and controls. For the multilevel regression analyses (“predictor modeling”), we included an observed predictor for the random effects at the between level, including individual mood disorder subtypes compared to controls and to each other, adjusted for age and sex (binary self‐reported). Herein, we present unstandardized results, which allow for an interpretation close to the original scales that were used in measuring the variables; this is salient given the practicality of interpreting a scale that ranges from 1 to 7 with defined anchors. Foundational syntax is publicly available (Hamaker & Muthen, 2021; Hamaker et al., 2018); our models are closely aligned with Multilevel Model 2 (stratified) and Multilevel Model 3 (predictors) described by Hamaker and colleagues (Hamaker & Muthen, 2021; Hamaker et al., 2018). Further, we present sample syntax for our stratified models in the Supporting Information Methods.

## 2 RESULTS

### 2.1 Sample characteristics

Three‐hundred sixty‐two participants with BP1 (*n* = 56), BPII (*n* = 54), MDD (*n* = 156), or no psychiatric disorder (controls; *n* = 96) had EMA data (Table 1). The average age was 43.1 years (standard deviation, 18.1; range: 11–85). Most participants reported their racial or ethnic identity as White; however, mood disorder and control groups were not different in their composition of racial or ethnic identities. Approximately 63% of the sample was female. There were more females (67.7%; *p* = .001) than males among persons with mood disorders, whereas the control group composition was balanced between males and females. Participants completed over 77% of mood prompts, yielding a total of 20,272 observations.

**TABLE 1:** Sample characteristics comparing participants with a major mood disorder or no disorder who have wave 1 EMA data in the NIMH Family Study.

	Total (*N* = 362)	Mood disorders (*N* = 266)	Controls (*N* = 96)
Age, mean (*SD*)	43.11 (18.10)	42.90 (16.92)	43.71 (21.12)
Sex, *n* (%)			
Male	135 (37.29)	86 (32.33)	49 (51.04)
Female	227 (62.71)	180 (67.67)	47 (48.96)
Self‐reported racial or ethnic identity, *n* (%)^a^			
White	289 (79.83)	212 (79.70)	77 (80.21)
Black or African American, Asian, American Indian, Alaska Native, more than one race, or other	60 (16.57)	44 (16.54)	16 (16.67)
Not reported	13 (3.59)	–^a^	–^a^
Lifetime DSM‐IV diagnoses, *n* (%)			
Bipolar‐I	56 (15.47)	56 (21.05)	–
Bipolar‐II	54 (14.92)	54 (20.30)	–
Major depressive disorder	156 (43.09)	156 (58.65)	–
Anxiety disorder	209 (57.73)	209 (78.57)	–
Substance use disorder	76 (21.05)	76 (28.68)	–
None	96 (26.52)	–	96 (100)

*Note*: Anxiety disorders included agoraphobia, obsessive–compulsive disorder, generalized anxiety disorder, panic disorder, posttraumatic stress disorder, separation anxiety, social anxiety, and specific phobia. Anxiety and substance use disorders are not mutually exclusive, and therefore could be comorbid with a mood disorder.

^a^ Self‐reported racial or ethnic identities other than White were combined here to protect participant privacy and prevent re‐identification due to small cell sizes. Numbers of participants who did not report a racial or ethnic identity are also not presented by mood group for privacy reasons.

### 2.2 Affective dynamics in mood disorder subtypes

To synthesize findings and aid interpretation, Figure S1 visualizes directionality and significance of within‐stratum effect estimates (Table 2), and directionality and significance of pairwise differences between diagnostic groups from predictor models (bipolar subtypes compared to each other and controls [Table 3]; major mood disorder categories BD and MDD compared to each other and controls [Table 4]).

**TABLE 2:** Point estimates (posterior means) and 95% credible intervals for means of affective dynamics at the between‐person level, stratified by diagnostic group.

	Bipolar‐I (*n* = 56)			Bipolar‐II (*n* = 54)			Bipolar disorder (*n* = 110)			Major depressive disorder (*n* = 156)			Controls (*n* = 96)		
	Mean	L2.5	U2.5	Mean	L2.5	U2.5	Mean	L2.5	U2.5	Mean	L2.5	U2.5	Mean	L2.5	U2.5
Individual mean level															
Sad, *μ*_S,_ *_i_*	**3.121**	2.244	4.04	**2.963**	2.086	3.798	**3.039**	2.819	3.258	**2.842**	2.668	3.017	**2.389**	2.148	2.631
Anxious, *μ*_A,_ *_i_*	**2.799**	1.865	3.705	**2.802**	1.713	3.807	**2.782**	2.54	3.031	**2.554**	2.378	2.737	**2.036**	1.82	2.264
Active, *μ*_V,_ *_i_*	**3.11**	2.25	3.913	**3.183**	2.334	3.968	**3.092**	2.892	3.294	**3.163**	3.008	3.32	**3.436**	3.17	3.721
Energetic, *μ*_E,_ *_i_*	**3.437**	2.678	4.141	**3.499**	2.766	4.253	**3.462**	3.281	3.642	**3.618**	3.462	3.766	**4.057**	3.817	4.293
Inertia															
Sad, *φ*_SS,_ *_i_*	0.221	−0.048	0.482	0.168	−0.143	0.467	**0.203**	0.131	0.275	**0.172**	0.119	0.224	**0.13**	0.044	0.221
Anxious, *φ*_AA,_ *_i_*	0.109	−0.124	0.346	0.179	−0.141	0.482	**0.147**	0.079	0.215	**0.15**	0.1	0.205	**0.154**	0.08	0.232
Active, *φ*_VV,_ *_i_*	0.1	−0.141	0.325	0.096	−0.204	0.377	**0.12**	0.047	0.193	**0.063**	0.014	0.112	0.02	−0.065	0.108
Energetic, *φ*_EE,_ *_i_*	0.113	−0.109	0.321	0.091	−0.196	0.399	**0.106**	0.042	0.169	**0.101**	0.054	0.147	**0.151**	0.076	0.226
Cross‐lag															
Anxious → Sad, *φ*_SA,_ *_i_*	0.001	−0.194	0.198	0.044	−0.232	0.337	0.022	−0.033	0.081	**0.051**	0.009	0.095	0.071	−0.009	0.152
Active → Sad, *φ*_SV,_ *_i_*	−0.025	−0.176	0.132	0.007	−0.237	0.268	−0.017	−0.068	0.033	−0.011	−0.048	0.026	0.004	−0.044	0.005
Energetic → Sad, *φ*_SE,_ *_i_*	−0.01	−0.166	0.143	−0.007	−0.267	0.238	−0.004	−0.054	0.047	−0.014	−0.051	0.025	−0.032	−0.09	0.025
Sad → Anxious, *φ*_AS,_ *_i_*	0.084	−0.183	0.35	0.08	−0.224	0.419	**0.079**	0.005	0.15	**0.0**	0.02	0.143	0.036	−0.047	0.115
Active → Anxious, *φ*_AV,_ *_i_*	0.03	−0.158	0.214	0.039	−0.245	0.334	0.034	−0.024	0.094	−0.01	−0.05	0.024	−0.033	−0.086	0.021
Energetic → Anxious, *φ*_AE,_ *_i_*	−0.009	−0.219	0.195	−0.013	−0.294	0.264	−0.012	−0.072	0.045	0.012	−0.031	0.055	0	−0.057	0.005
Sad → Active, *φ*_VS,_ *_i_*	0.1	−0.369	0.638	0.068	−0.413	0.539	0.087	−0.021	0.206	0.004	−0.062	0.071	0.104	−0.024	0.251
Anxious → Active, *φ*_VA,_ *_i_*	0.1	−0.287	0.569	0.078	−0.287	0.436	**0.1**	0.001	0.212	0.041	−0.026	0.11	−0.052	−0.206	0.081
Energetic → Active, *φ*_VE,_ *_i_*	0.002	−0.226	0.249	0.016	−0.302	0.336	0.006	−0.062	0.074	0.045	−0.007	0.095	0.084	−0.006	0.17
Sad → Energetic, *φ*_ES,_ *_i_*	0.12	−0.233	0.529	0.007	−0.417	0.443	0.068	−0.031	0.17	−0.019	−0.077	0.043	0.078	−0.025	0.188
Anxious → Energetic, *φ*_EA,_ *_i_*	−0.016	−0.316	0.315	0.066	−0.282	0.396	0.029	−0.056	0.122	−0.037	−0.098	0.021	**−0.156**	−0.294	−0.039
Active → Energetic, *φ*_EV,_ *_i_*	0.016	−0.211	0.231	0.007	−0.246	0.278	0.019	−0.043	0.082	−0.035	−0.08	0.009	−0.053	−0.122	0.018
Log variances of innovations															
Sad, log(*π*_S_)	−0.506	−1.655	0.595	−0.175	−0.77	0.414	**−0.333**	−0.579	−0.08	**−0.318**	−0.504	−0.125	**−0.745**	−1.119	−0.367
Anxious, log(*π*_A_)	−0.261	−1.504	0.865	0.121	−0.483	0.724	−0.059	−0.323	0.213	−0.0	−0.298	0.093	**−0.776**	−1.248	−0.33
Active, log(*π*_V_)	0.391	−0.431	1.155	0.439	−0.228	1.08	**0.449**	0.27	0.621	**0.414**	0.288	0.539	**0.371**	0.17	0.562
Energetic, log(*π*_E_)	0.312	−0.41	0.991	0.302	−0.266	0.872	**0.336**	0.17	0.494	**0.142**	0.009	0.273	0.066	−0.132	0.272

*Note*: Bold means are those that are estimated to differ from 0 based on the bounds of the 95% credible interval. Bipolar disorder is a combined variable of individuals with either bipolar‐I or bipolar‐II. Each column of means with its corresponding credible interval is from its unique multivariate stratified model, with estimates that simultaneously adjust for all dynamics including individual mean levels, inertias, cross‐regressions, and innovation variances.

Abbreviations: L2.5, lower 2.5% of credible interval; U2.5, upper 2.5% of credible interval.

**TABLE 3:** Regression coefficients and 95% credible intervals for predictor modeling testing differences in affective dynamics between bipolar disorder subtypes and controls.

	Bipolar‐I vs. controls (*n* = 152)			Bipolar‐II vs. controls (*n* = 150)			Bipolar‐I vs. bipolar‐II (*n* = 110)		
	Est.	L2.5	U2.5	Est.	L2.5	U2.5	Est.	L2.5	U2.5
Individual mean level									
Sad, *μ*_S,_ *_i_*	**0.339**	0.195	0.49	**0.298**	0.167	0.427	0.039	−0.145	0.214
Anxious, *μ*_A,_ *_i_*	**0.356**	0.215	0.501	**0.371**	0.235	0.506	−0.02	−0.213	0.177
Active, *μ*_V,_ *_i_*	**−0.16**	−0.308	−0.006	−0.11	−0.247	0.034	−0.05	−0.218	0.112
Energetic, *μ*_E,_ *_i_*	**−0.272**	−0.407	−0.136	**−0.239**	−0.365	−0.112	−0.017	−0.175	0.129
Inertia									
Sad, *φ*_SS,_ *_i_*	**0.056**	0.012	0.1	0.026	−0.018	0.072	0.003	−0.047	0.058
Anxious, *φ*_AA,_ *_i_*	**−0.036**	−0.074	−0.001	0.028	−0.013	0.07	−0.044	−0.091	0.004
Active, *φ*_VV,_ *_i_*	**0.059**	0.017	0.101	0.026	−0.015	0.067	0.037	−0.012	0.082
Energetic, *φ*_EE,_ *_i_*	−0.033	−0.07	0.004	−0.029	−0.07	0.012	0.003	−0.039	0.044
Cross‐lag									
Anxious → Sad, *φ*_SA,_ *_i_*	**−0.049**	−0.083	−0.016	−0.023	−0.06	0.013	−0.005	−0.044	0.033
Active → Sad, *φ*_SV,_ *_i_*	−0.013	−0.028	0.004	0.001	−0.024	0.025	−0.018	−0.043	0.009
Energetic → Sad, *φ*_SE,_ *_i_*	0.009	−0.01	0.027	0.005	−0.021	0.029	0.003	−0.029	0.033
Sad → Anxious, *φ*_AS,_ *_i_*	0.031	−0.008	0.072	0.007	−0.034	0.052	0.004	−0.045	0.056
Active → Anxious, *φ*_AV,_ *_i_*	0.009	−0.01	0.03	0.022	−0.004	0.049	−0.013	−0.046	0.022
Energetic → Anxious, *φ*_AE,_ *_i_*	0.004	−0.017	0.023	−0.009	−0.038	0.021	0.009	−0.027	0.046
Sad → Active, *φ*_VS,_ *_i_*	−0.008	−0.064	0.048	−0.014	−0.063	0.035	0.016	−0.049	0.089
Anxious → Active, *φ*_VA,_ *_i_*	0.025	−0.021	0.071	**0.044**	0.001	0.085	−0.022	−0.07	0.027
Energetic → Active, *φ*_VE,_ *_i_*	**−0.045**	−0.084	−0.008	−0.029	−0.07	0.01	−0.018	−0.059	0.025
Sad → Energetic, *φ*_ES,_ *_i_*	−0.009	−0.049	0.037	−0.027	−0.072	0.019	0.034	−0.021	0.087
Anxious → Energetic, *φ*_EA,_ *_i_*	0.019	−0.023	0.06	**0.059**	0.022	0.097	**−0.052**	−0.098	−0.007
Active → Energetic, *φ*_EV,_ *_i_*	**0.048**	0.018	0.081	0.024	−0.01	0.058	0.025	−0.014	0.061
Log variances of innovations									
Sad, log(*π*_S_)	0.085	−0.13	0.283	**0.283**	0.101	0.465	**−0.208**	−0.39	−0.019
Anxious, log(*π*_A_)	0.236	−0.018	0.474	**0.443**	0.235	0.651	**−0.208**	−0.396	−0.01
Active, log(*π*_V_)	0.03	−0.084	0.145	0.016	−0.081	0.113	0.009	−0.114	0.14
Energetic, log(*π*_E_)	**0.126**	0.023	0.23	0.081	−0.012	0.173	0.032	−0.09	0.149

*Note*: Bold effect estimates are those that are estimated to differ from 0 based on the bounds of the 95% credible interval. Each column of effect estimates (regression coefficients) is from a unique, multivariate pairwise model that simultaneously adjusts for all dynamics including individual mean levels, inertias, cross‐regressions, and innovation variances. All models adjust for age and sex.

Abbreviations: Est., effect estimate; L2.5, lower 2.5% of credible interval; U2.5, upper 2.5% of credible interval.

**TABLE 4:** Regression coefficients and 95% credible intervals for predictor modeling testing differences in affective dynamics between major mood disorder categories and controls.

	Bipolar disorder vs. controls (*n* = 206)			Bipolar disorder vs. major depressive disorder (*n* = 266)			Major depressive disorder vs. controls (*n* = 252)		
	Est.	L2.5	U2.5	Est.	L2.5	U2.5	Est.	L2.5	U2.5
Individual mean level									
Sad, *μ*_S,_ *_i_*	**0.33**	0.21	0.449	0.084	−0.024	0.19	**0.248**	0.142	0.357
Anxious, *μ*_A,_ *_i_*	**0.383**	0.255	0.506	0.106	−0.007	0.217	**0.275**	0.166	0.385
Active, *μ*_V,_ *_i_*	**−0.137**	−0.264	−0.015	−0.011	−0.116	0.093	−0.111	−0.224	0
Energetic, *μ*_E,_ *_i_*	**−0.267**	−0.374	−0.157	−0.061	−0.155	0.035	**−0.183**	−0.286	−0.081
Inertia									
Sad, *φ*_SS,_ *_i_*	**0.051**	0.013	0.09	0.015	−0.015	0.046	**0.037**	0.005	0.069
Anxious, *φ*_AA,_ *_i_*	−0.013	−0.046	0.021	−0.001	−0.032	0.028	−0.014	−0.044	0.017
Active, *φ*_VV,_ *_i_*	**0.047**	0.01	0.083	**0.031**	0	0.06	0.017	−0.015	0.048
Energetic, *φ*_EE,_ *_i_*	−0.031	−0.063	0.001	−0.001	−0.028	0.026	**−0.029**	−0.056	0
Cross‐lag									
Anxious → Sad, *φ*_SA,_ *_i_*	**−0.045**	−0.075	−0.014	−0.013	−0.035	0.01	**−0.032**	−0.058	−0.005
Active → Sad, *φ*_SV,_ *_i_*	−0.009	−0.024	0.008	−0.011	−0.028	0.006	0.001	−0.014	0.017
Energetic → Sad, *φ*_SE,_ *_i_*	0.009	−0.008	0.026	0.006	−0.012	0.024	0.003	−0.013	0.019
Sad → Anxious, *φ*_AS,_ *_i_*	0.032	−0.004	0.064	0.005	−0.027	0.039	0.027	−0.008	0.059
Active → Anxious, *φ*_AV,_ *_i_*	0.014	−0.004	0.035	0.006	−0.013	0.026	0	−0.015	0.016
Energetic → Anxious, *φ*_AE,_ *_i_*	0.001	−0.018	0.02	0.002	−0.02	0.022	0.006	−0.011	0.022
Sad → Active, *φ*_VS,_ *_i_*	−0.005	−0.05	0.042	0.03	−0.007	0.067	−0.034	−0.07	0.006
Anxious → Active, *φ*_VA,_ *_i_*	0.029	−0.01	0.071	−0.001	−0.035	0.029	0.027	−0.011	0.064
Energetic → Active, *φ*_VE,_ *_i_*	**−0.038**	−0.07	−0.006	−0.022	−0.048	0.006	−0.015	−0.045	0.014
Sad → Energetic, *φ*_ES,_ *_i_*	−0.012	−0.051	0.026	**0.042**	0.011	0.074	**−0.047**	−0.081	−0.015
Anxious → Energetic, *φ*_EA,_ *_i_*	**0.036**	0.001	0.071	−0.007	−0.035	0.021	**0.043**	0.013	0.074
Active → Energetic, *φ*_EV,_ *_i_*	**0.039**	0.011	0.067	**0.033**	0.009	0.056	0.008	−0.017	0.033
Log variances of innovations									
Sad, log(*π*_S_)	**0.189**	0.024	0.348	0.003	−0.119	0.122	**0.191**	0.049	0.337
Anxious, log(*π*_A_)	**0.341**	0.161	0.518	0.027	−0.106	0.149	**0.323**	0.159	0.491
Active, log(*π*_V_)	0.021	−0.071	0.113	0.015	−0.062	0.098	0.008	−0.073	0.09
Energetic, log(*π*_E_)	**0.106**	0.026	0.189	**0.081**	0.005	0.158	0.016	−0.064	0.096

*Note*: Bold effect estimates are those that are estimated to differ from 0 based on the bounds of the 95% credible interval. Each column of effect estimates (regression coefficients) is from a unique, multivariate pairwise model that simultaneously adjusts for all dynamics including individual mean levels, inertias, cross‐regressions, and innovation variances. All models adjust for age and sex.

Abbreviations: Est., effect estimate; L2.5, lower 2.5% of credible interval; U2.5, upper 2.5% of credible interval.

#### 2.2.1 Individual mean level

All mood subtypes had higher average sad and anxious ratings and lower average energetic ratings compared to controls. Only individuals with BD, likely driven by effects in the BPI subtype, had lower mean active levels compared to controls. For example, the within‐strata patterns for average active level in BPI (3.110, 95% CI: 2.25–3.913), BD (3.092, 95% CI: 2.892–3.294), and controls (3.436, 95% CI: 3.17–3.721) (Table 2) were further reflected in the significant adjusted regression coefficients (“Est.”) comparing BPI to controls (Est.: −0.160, 95% CI: −0.308 to 0.006) and BD to controls (Est.: −0.137, 95% CI: −0.264 to 0.015) (Tables 3 and 4, respectively)—differences that were not significant in BPII or MDD. Variances for the individual mean levels of all affective states ranged from 0.840 to 1.753; considering the raw scale of 1–7, this provides evidence of between‐person heterogeneity in individual means, especially among controls (Table S1, right).

#### 2.2.2 Inertia

We found positive inertia for almost all affective states within strata, indicating a tendency for increases in affective states to carry over from one timepoint to the next, with the exception of active among controls (Table 2). Compared to controls, BD (Est.: 0.051, 95% CI: 0.013–0.09)—likely driven by BPI (Est.: 0.056, 95% CI: 0.012–0.100)—and MDD (Est.: 0.037, 95% CI: 0.005–0.069) had significantly higher sadness inertia, suggesting that when sadness increased from individual mean levels, the heightened sadness persisted (Tables 3 and 4). In contrast, BPI had significantly lower anxious inertia than controls (Est.: −0.036, 95% CI: −0.074 to –0.001). BD was associated with higher active inertia compared to both MDD (Est.: 0.031, 95% CI: 0–0.060) and controls (Est.: 0.047, 95% CI: 0.01–0.083), which again may have been driven by the BPI subtype (Est.: 0.059, 95% CI: 0.017–0.101). Thus, increases above individual mean sadness and activation were more likely to perpetuate, and anxiety less so, among individuals with BPI. Finally, compared to controls, MDD was associated with lower energetic inertia (Est.: −0.029, 95% CI: −0.056 to 0).

#### 2.2.3 Cross‐lags

The groups displayed different patterns of cross‐lags across affective states. Within stratum, persons with BD had positive cross‐lags from sad to anxious (0.079, 95% CI: 0.005–0.150) and anxious to active (0.100, 95% CI: 0.001–0.212), MDD exhibited a feedback loop from anxious to sad (0.051, 95% CI: 0.009–0.095) and sad to anxious (0.086, 95% CI: 0.028–0.143), and controls displayed a negative cross‐lag from anxious to energetic (−0.156, 95% CI: −0.294 to –0.039) (Table 2). Although both BD and MDD reported increased anxiety subsequent to prior sadness within strata, this spillover was not significantly different compared to controls or each other. In contrast, compared to controls, both BD, likely driven by BPI (Est.: −0.049, 95% CI: −0.083 to –0.016), and MDD were associated with significantly less spillover from anxious to sad (Tables 3 and 4). However, increased activation subsequent to prior increased anxiety was significantly more prominent in BPII compared to controls (BPII mean 0.078 vs. controls mean −0.057; Est.: 0.044, 95% CI: 0.001–0.085).

Interestingly, cross‐reactivity between energetic ratings and activation was largely null, or neutral, in BD. In contrast, controls reported significantly more activation following increased energy compared to BD, especially BPI (BPI vs. controls Est.: −0.045, 95% CI: −0.084 to –0.008), while increased activation blunted subsequent energy. Additionally, compared to BD, those with MDD reported lower energy after increased activation. Though modest, these effects were qualitatively different: higher spillover from active to energetic associated with BD compared to both controls (Est.: 0.039, 95% CI: 0.011–0.067) and MDD (Est.: 0.033, 95% CI: 0.009–0.056) may suggest that increased activation failing to deplete energy may be unique to BPI (Est.: 0.048, 95% CI: 0.018–0.081) and more generally, independence of activation from energy in BPI. Sadness was also significantly associated with subsequently blunted energy for those with MDD compared to both BD and controls, and anxiety was significantly associated with subsequently blunted energy for controls compared to MDD and BD, particularly BPII.

#### 2.2.4 Innovation variances

In predictor modeling, both BD—likely driven by BPII—and MDD were associated with significantly larger innovation variances of sad and anxious states, suggesting higher intensity of changes around individual mean levels, or lability, compared to controls (Tables 3 and 4). For example, this corresponded with anxious innovation variances of 0.943 (95% CI: 0.724–1.237), 0.906 (95% CI: 0.742–1.097), and 0.460 (95% CI: 0.287–0.719) for BD, MDD, and controls, respectively (Table 2 values exponentiated). Within BD subtypes, BPII had significantly higher lability of sad and anxious than BPI. Additionally, BD was significantly associated with higher lability of energy compared to MDD and controls, with the latter association likely being driven by BPI. These correspond to energy innovation variances of 1.399 (95% CI: 1.185–1.639) for BD, 1.153 (95% CI: 1.009–1.314) for MDD, and 1.068 (95% CI: 0.876–1.313) for controls (again exponentiated). While significant within‐person lability of active levels was observed within BD, MDD, and control strata, there were no group differences in activation innovation variances in predictor modeling.

## 3 DISCUSSION

We used DSEM to develop finer grained characterization of affective dynamics in mood disorders. We found that people with mood disorders displayed higher levels of sad and anxious mood and lower levels of energy as compared to controls. People with BPI had lower average activation, and lower energy that was independent of activation, compared to MDD or controls. However, increases in activation were significantly more likely to perpetuate in those with BPI. Those with BPII were characterized by higher lability of sad and anxious mood compared to BPI and controls but not MDD. Compared to BD and controls, those with MDD exhibited cross‐augmentation of sadness and anxiety, and sadness blunted energy.

Although the aforementioned average levels of higher sadness and anxiousness and lower energy were consistent across mood subtypes compared to controls, lower active levels were likely specific to BPI. Prior evidence is mixed with respect to BD and average levels of “positive affect” (Aan Het Rot et al., 2012; Gruber et al., 2013; Knowles et al., 2007). This finding may be due to our assessing pure activation in its own dimension rather than crossed with valence. Specifically, nomenclature and measures of “positive” and “negative” affect build on Watson and Tellegen's (1985) work. That nomenclature is somewhat misleading because the constructs of negative and positive affect cross octants—that is, they involve both valence and arousal/activation (Larsen & Diener, 1992). Doing so may introduce measurement error and inhibit understanding of specificity. Previous findings suggest the centrality of energy and activity dysregulation in BD (Cheniaux et al., 2014), the salience of lower mean activity in euthymic BD (Cheniaux et al., 2014; Merikangas et al., 2019; Scott et al., 2017), and the importance of disentangling valence from activation. Previous findings also showed remitted BD and MDD groups aligned in reporting greater negative affect as well as sad mood measured individually compared to controls. The present study is larger than prior work, which may have allowed us to identify some differences between mood disorder subtypes not previously observed (Gruber et al., 2013; Lamers et al., 2018; Knowles et al., 2007; Scott et al., 2020). Moreover, by using DSEM, we are the first to have included all affective dynamics and circumplex dimensions in the same model, which may attenuate all but the most robust differences between mood disorder subtypes. Replication is needed.

Affect broadly tended to carry over from one timepoint to the next but the tendency for sadness inertia was significantly stronger in BPI and MDD. Elevation in activation was significantly more likely to perpetuate in BD, especially BPI, for whom anxiety was fleeting. In contrast, energetic feelings were less likely to perpetuate for those with MDD compared to controls. Our findings of greater sadness inertia and lower anxiety inertia in BPI differ from Lamers et al. (2018), who did not find significant differences between mood or anxiety groups in sad and anxious inertia examined univariately. Some participants from that earlier report (Lamers et al., 2018) are analyzed herein. However, our modeling approaches are quite different (univariate vs. multivariate)—reflecting the recent statistical advancements of this field, our sample has grown meaningfully larger, we did not include persons with anxiety disorders but no mood disorders, and we expanded our scope to the full emotional circumplex. Our finding of the tendency for increased activation to perpetuate over time in BD is complementary with decades of work on the behavioral activation/approach system as a potential characteristic or prognosticator of BD (Alloy & Abramson, 2010; Johnson et al., 2012).

One of the only other studies to use DSEM to examine affective dynamics did not find evidence of cross‐lags, that is, augmenting or blunting effects, between positive and negative affect in internalizing disorders (Scott et al., 2020); their sample was smaller and young, and the use of summary scales may have obscured more subtle fluctuations. We found that those with MDD experienced a feedback loop of cross‐reactivity between sad and anxious levels, as well as a drop in energy after increased sadness. This is important when considering the significant tendency for sadness to persist, which may then amplify anxiety and, in turn, precede additional increased sadness. These findings may provide real‐time evidence of negative attentional biases and cognitive styles, even in euthymia, that have been considered important in understanding mechanisms of recurrence in MDD and anxiety disorders (Peckham et al., 2010). Increased anxiety blunted subsequent energy in controls relative to MDD and BPII, and augmented sadness relative to BPI. Anxiety demonstrated starkly different cross‐reactivity in BPII, augmenting subsequent energy and activation. Another qualitative difference in cross‐reactivity of emotions was between energy and activation in BPI. Compared to BD, increased activation blunted subsequent energy in MDD and controls and energy augmented activation for controls. These patterns were likely driven by BPI, for whom activation, though self‐perpetuating, did not spill over into energy, and energy did not spill over into activation. More research is therefore needed on mechanistic drivers of energy and activation, *disambiguated*, in BPI.

Higher innovation variances of sad and anxious across mood groups—likely driven by BPII and MDD—suggest greater within‐person variation from timepoint to timepoint, or more affective lability. These findings were mostly consistent with Lamers et al. (2018), although we tested differences between BD subtypes and were therefore able to detect lower sad and anxious lability in BPI relative to BPII. The higher instability of “negative emotions” confirms prior studies of symptom patterns of people with BD (Houben et al., 2015; Scott et al., 2020). Within‐person lability in energy was significantly larger in BD, likely driven by BPI, compared to both MDD and controls. Previous meta‐analysis has demonstrated an association between BD and variability of “positive emotions” (Houben et al., 2015), so it may be that energy, a product of pleasant valence and higher activation, is one specific example of such.

An important caveat is the subtlety of mood fluctuations; overall, emotional ratings trended toward somewhat happy, mostly calm, somewhat inactivated/quiet, and neutral or balanced with respect to energy. Additionally, *higher sadness* might alternatively be labeled *lower happiness*. Despite these subtleties, a distinct picture emerged, such as higher unpleasant valence across mood groups that was more persistent within and among affective states, and also displayed higher intensity of changes (lability). The findings of a recent meta‐analysis of adolescents and young adults showed that high instability and variability of negative affect were associated with more depressive symptoms among individuals with low average negative affect and fewer depressive symptoms among those with high average negative affect (Maciejewski et al., 2022). In combination with our findings, this raises the question of whether variation and cross‐reactivity in affect can at times serve as a potential protective “mechanism” or perhaps simply as an indicator/*trait* of reduced dysregulation—both a “lifting” from more challenging emotions and a drive to return to homeostasis of balanced emotions.

### 3.1 Limitations with offsetting strengths and future directions

As participants were not in‐episode for this study, we did not take into account the recency or severity of episodes on affective dynamics, focusing instead on lifetime diagnoses for this cross‐sectional analysis. Additionally, over time, some participants without a diagnosis at the time of EMA (e.g., adolescents or young adults) may eventually develop a psychiatric disorder. However, our findings are focused on the relationship between current affective dynamics and history of a disorder up until the time of EMA, and we do not attempt to predict future trajectories. Such features and trajectories of disorder status may be a promising future direction for investigations of mood transitions that incorporate intensive longitudinal data in longitudinal cohorts. Two weeks is not long enough to cover full hormone cycles in females, which are known to be linked to changes in energy and mood among other physiology (Slyepchenko et al., 2021). Investigators and clinicians interested in uncovering sex differences in fundamental affective dynamics of mood disorders as well as their interrelationships with biologic influences should sample experiences over longer periods. Timepoints were treated equally based on wakeful hours for interpretability, which may affect strength of association (Asparouhov et al., 2017). Additionally, interpretation of findings from interval‐contingent schedules of roughly equal time periods may be different from fully random signal‐dependent schedules (Hall et al., 2021). We also did not examine within‐day versus between‐day fluctuation in affect, which may differ for positive affect in individuals with high hypomanic scores (Sperry & Kwapil, 2022); future investigations will quantify the impact of sleep on continuation versus interruption of affective dynamics.

Although theoretically informed, there is not an objective truth to which these findings can be validated, and words for emotions are not the same thing as emotions (Larsen & Diener, 1992). However, a circumplex framework for understanding affect and emotional dynamics has been well‐studied in clinical and nonclinical samples. Our findings build upon that tradition and make use of state‐of‐the‐science modeling to understand real‐time variability of individual emotions and their cross‐reactivity, simultaneously adjusted for each other, in a richly phenotyped community‐based sample, which is a key advancement in understanding affective dynamics of mood disorders. Although we statistically adjusted for age in our models, future work should directly interrogate within‐person change in depth of emotional expression longitudinally in persons with mood disorders as well as in larger samples of youth to better understand how *development* may impact measurement of emotion using the circumplex (Posner et al., 2005). There are other approaches to understanding affective dynamics—positive and negative affect being predominant. However, for this initial novel application of DSEM characterizing dynamics in mood disorder subtypes, we did not believe the solution to understanding the complexity of the emotional circumplex was to subsume individual emotions under scales that cross octants. Future research should examine the utility of individual octants or dimensions in contrast to data‐reducing approaches such as scales or common latent factors in understanding differences across mood disorders (Bos et al., 2022; Taylor et al., 2021) and general affective disturbance and risk in nonclinical samples (Sperry & Kwapil, 2022; Trampe et al., 2015). Finally, we did not examine what is *driving* these fluctuations and thus cannot determine whether they are stochastic, innate, or sparked by health behaviors or comorbidities, for example (Alloy & Abramson, 2010; Johnson et al., 2012; Lewis et al., 2022; Pemberton & Fuller Tyszkiewicz, 2016; Quick et al., 2022; Stapp et al., 2022). Our perspective is that there is inherent worth in understanding these characteristics and dynamics as they are, irrespective of putative cause. Future research on multiple health and social behaviors and contexts simultaneously with multivariate affective dynamics in real time would enrich understanding of antecedents and intervention points.

## 4 CONCLUSION

These findings demonstrate differences in the affective dynamics of BD compared to MDD between episodes. Future studies should examine the stability of the patterns across episodes that may then serve as early warning signals of upcoming mood transitions (Bos et al., 2022; Van De Leemput et al., 2014; Yee et al., 2021). Given the exploratory nature of this approach, future work should seek to independently replicate these findings and assess their readiness for clinical applications (Bosley et al., 2019) such as digital feedback, therapy and psychoeducation, or pharmacology. Whether said dynamics can be fully operationalized, predicted, and (self)regulated will have significant clinical import. Herein, we have demonstrated DSEM's utility in multivariate characterization of dynamic affect, finding that mood disorder subtypes are differentiated by distinctive patterns of affective dynamics, which may deepen our understanding of mood disorders.

## CONFLICT OF INTEREST STATEMENT

The authors declare no conflicts of interest.

### PEER REVIEW

The peer review history for this article is available at https://publons.com/publon/10.1002/brb3.3134.

## Supporting information

Figure S1. Directionality of effects of affective dynamics in and between diagnostic subgroups

Supporting Information

Supplemental Table 1. Point estimates (posterior means) and variances with 95% credible intervals of affective dynamics, stratified by diagnostic group, alongside re‐runs of each model with at least 10,000 iterations

Supplemental Table 2. Regression coefficients and 95% credible intervals for predictor modeling testing the association of mood disorder subtypes with affective dynamics, alongside re‐runs of each model with at least 10,000 iterations

## Data Availability

Certain data are available on request; data are not publicly available due to privacy.
