# Supplementary material for: Specificity of affective dynamics of bipolar and major depressive disorder
Source: Brain Behav. 2023 Aug 13;13(9):e3134. doi: 10.1002/brb3.3134 (PMC10498074; doi:10.1002/brb3.3134)
Supplement: Supplementary file 1 — Figure S1. Directionality of effects of affective dynamics in and between diagnostic subgroups [file BRB3-13-e3134-s002.pdf]

Figure S1. Directionality of effects of affective dynamics in and between diagnostic subgroups

	Comparisons of Bipolar Subtypes									Comparisons of Major Mood Disorder Categories								
	**BPI vs Controls** (n=152)			**BPII vs Controls** (n=150)			**BPI vs BPII** (n=110)			**BD vs Controls** (n=206)			**BD vs MDD** (n=266)			**MDD v Controls** (n=252)		
	BPI	Control	*Delta:* BPI Higher (+) or Lower (-) than Control	BPII	Control	*Delta:* BPII Higher (+) or Lower (-) than Control	BPI	BPII	*Delta:* BPI Higher (+) or Lower (-) than BPII	BD	Control	*Delta:* BD Higher (+) or Lower (-) than Control	BD	MDD	*Delta:* BD Higher (+) or Lower (-) than MDD	MDD	Control	*Delta:* MDD Higher (+) or Lower (-) than Control
**Individual Mean Level**																		
Sad, $\mu_{S,i}$	**+**	**+**	**+**	**+**	**+**	**+**	**+**	**+**	+	**+**	**+**	**+**	**+**	**+**	+	**+**	**+**	**+**
Anxious, $\mu_{A,i}$	**+**	**+**	**+**	**+**	**+**	**+**	**+**	**+**	-	**+**	**+**	**+**	**+**	**+**	+	**+**	**+**	**+**
Active, $\mu_{V,i}$	**+**	**+**	**-**	**+**	**+**	-	**+**	**+**	-	**+**	**+**	**-**	**+**	**+**	-	**+**	**+**	-
Energetic, $\mu_{E,i}$	**+**	**+**	**-**	**+**	**+**	**-**	**+**	**+**	-	**+**	**+**	**-**	**+**	**+**	-	**+**	**+**	**-**
**Inertia**																		
Sad, $\varphi_{SS,i}$	+	**+**	**+**	+	**+**	+	+	+	+	**+**	**+**	**+**	**+**	**+**	+	**+**	**+**	**+**
Anxious, $\varphi_{AA,i}$	+	**+**	**-**	+	**+**	+	+	+	-	**+**	**+**	-	**+**	**+**	-	**+**	**+**	-
Active, $\varphi_{VV,i}$	+	+	**+**	+	+	+	+	+	+	**+**	+	**+**	**+**	**+**	**+**	**+**	+	+
Energetic, $\varphi_{EE,i}$	+	**+**	-	+	**+**	-	+	+	+	**+**	**+**	-	**+**	**+**	+	**+**	**+**	**-**
**Cross-Lag**																		
Anxious → Sad, $\varphi_{SA,i}$	+	+	**-**	+	+	-	+	+	-	+	+	**-**	+	**+**	-	**+**	+	**-**
Active → Sad, $\varphi_{SV,i}$	-	+	-	+	+	+	-	+	-	-	+	-	-	-	-	-	+	-
Energetic → Sad, $\varphi_{SE,i}$	-	-	+	-	-	+	-	-	-	-	-	+	-	-	+	-	-	+
Sad → Anxious, $\varphi_{AS,i}$	+	+	+	+	+	+	+	+	+	**+**	+	+	**+**	**+**	-	**+**	+	+
Active → Anxious, $\varphi_{AV,i}$	+	-	+	+	-	+	+	+	-	+	-	+	+	-	+	-	-	+
Energetic → Anxious, $\varphi_{AE,i}$	-	0	-	-	0	-	-	-	+	-	0	-	-	+	-	+	0	+
Sad → Active, $\varphi_{VS,i}$	+	+	-	+	+	-	+	+	+	+	+	-	+	+	+	+	+	-
Anxious → Active, $\varphi_{VA,i}$	+	-	+	+	-	**+**	+	+	+	**+**	-	+	**+**	+	+	+	-	+
Energetic → Active, $\varphi_{VE,i}$	+	+	**-**	+	+	-	+	+	-	+	+	**-**	+	+	-	+	+	-
Sad → Energetic, $\varphi_{ES,i}$	+	+	+	+	+	-	+	+	+	+	+	-	+	-	**+**	-	+	**-**
Anxious → Energetic, $\varphi_{EA,i}$	-	**-**	+	+	**-**	**+**	-	+	**-**	+	**-**	**+**	+	-	+	-	**-**	**+**
Active → Energetic, $\varphi_{EV,i}$	+	-	**+**	+	-	+	+	+	+	+	-	**+**	+	-	**+**	-	-	+
Log **Variances of Innovations**																		
Sad, $log(\pi_S)$	-	**-**	+	-	**-**	**+**	-	-	**-**	**-**	**-**	**+**	**-**	**-**	+	**-**	**-**	**+**
Anxious, $log(\pi_A)$	-	**-**	+	+	**-**	**+**	-	+	**-**	-	**-**	**+**	-	-	+	-	**-**	**+**
Active, $log(\pi_V)$	+	**+**	+	+	**+**	+	+	+	+	**+**	**+**	+	**+**	**+**	+	**+**	**+**	+
Energetic, $log(\pi_E)$	+	+	**+**	+	+	+	+	+	+	**+**	+	**+**	**+**	**+**	**+**	**+**	+	+

Note: BD, Bipolar Disorder type I or type II composite variable; BPI, Bipolar Disorder type I; BPII, Bipolar Disorder type II; MDD, Major Depressive Disoder.
Each unique pairwise model presents the directionality, positive (+) or negative (-) of the group means for a given random effect for each of the two groups being compared, followed by their difference, adjusted for covariates. Bold, colored symbols are 'significant': for diagnostic group means, that suggests significantly different from zero, and for the differences between those group means (delta), significance is based on the significance of the diagnostic comparison variable in multivariate regressions that adjusted for age and sex. All effect estimates are simultaneously adjusted for all of the affective dynamics (group means, inertia, cross-regressions, and innovation variances.)
Example interpretation of a comparison between BPI and Controls: The first two columns of values were taken from the stratified models (Table 2). BPI had positive sadness inertia but it was not significantly different from zero, represented by a simple +, while Controls had positive sadness inertia that was significantly different from zero, represented by a blue bold +. The delta is a simple subtraction between those means, but whether or not that delta gets bolded/shaded is taken from the predictor modeling (Table 3) and therefore lets us know whether the difference between groups for a particular emotional dynamic is significant independent of all other dynamics as well as age and sex. Continuing with the sadness inertia example, we see that BPI had significantly higher sadness inertia compared to controls, represented by the blue bold +, adjusted for the effects of age and sex on the model.

Legend:

**+** (blue)	positive effect or difference, significant
**-** (yellow, dotted)	negative effect or difference, significant
+	positive effect or difference, not significant
-	negative effect or difference, not significant
