# Supplementary material for: Specificity of affective dynamics of bipolar and major depressive disorder
Source: Brain Behav. 2023 Aug 13;13(9):e3134. doi: 10.1002/brb3.3134 (PMC10498074; doi:10.1002/brb3.3134)
Supplement: Supplementary file 2 — Supporting Information [file BRB3-13-e3134-s001.docx]

Supplemental Methods

“Specificity of Affective Dynamics of Bipolar and Major Depressive Disorder”

**Sample Mplus Syntax for STRATIFIED MODEL (unconditional affective dynamics by diagnostic group)**

Title: Affective dynamics using FOUR momentary mood states from mood circumplex

NIMH Family Study of Affective Spectrum Disorders

Stratum: BD (i or ii)

Data:

File = w1withSecondary_onlyBP1andBP2.txt;

Variable:

NAMES = studyid PD2timep PDactv PDener PDsad PDanxi;

CLUSTER = studyid;

USEVARIABLES = PDsad PDanxi PDactv PDener;

LAGGED = PDsad(1) PDanxi(1) PDactv(1) PDener(1);

!lagged creates new variables e.g. PDsad&1, sadness at t-1

TINTERVAL = PD2timep(1);

MISSING = all(-999);

Analysis:

Type = TWOLEVEL RANDOM;

ESTIMATOR = BAYES;

PROC = 2; BITER = (3000);

BSEED = 2203; THIN = 10;

Model:

%WITHIN%

phi_ss | PDsad ON PDsad&1; !Random slope for PDsad_t regressed on PDsad_t-1 (autoregression of sad)

beta_sa | PDsad ON PDanxi&1; !Random slope for PDsad_t regressed on PDanxi_t-1 (cross-lag from anxious to sad)

beta_sv | PDsad ON PDactv&1; !Random slope for PDsad_t regressed on PDactv_t-1 (cross-lag from active to sad)

beta_se | PDsad ON PDener&1; !Random slope for PDsad_t regressed on PDener_t-1 (cross-lag from energetic to sad)

phi_aa | PDanxi ON PDanxi&1; !Random slope for PDanxi_t regressed on PDanxi_t-1 (autoregression of anxious)

beta_as | PDanxi ON PDsad&1; !Random slope for PDanxi_t regressed on PDsad_t-1 (cross-lag from sad to anxious)

beta_av | PDanxi ON PDactv&1; !Random slope for PDanxi_t regressed on PDactv_t-1 (cross-lag from active to anxious)

beta_ae | PDanxi ON PDener&1; !Random slope for PDanxi_t regressed on PDener_t-1 (cross-lag from energetic to anxious)

phi_vv | PDactv ON PDactv&1; !Random slope for PDactv_t regressed on PDactv_t-1 (autoregression of active)

beta_vs | PDactv ON PDsad&1; !Random slope for PDactv_t regressed on PDsad_t-1 (cross-lag from sad to active)

beta_va | PDactv ON PDanxi&1; !Random slope for PDactv_t regressed on PDanxi_t-1 (cross-lag from anxious to active)

beta_ve | PDactv ON PDener&1; !Random slope for PDactv_t regressed on PDener_t-1 (cross-lag from energetic to active)

phi_ee | PDener ON PDener&1; !Random slope for PDener_t regressed on PDener_t-1 (autoregression of energetic)

beta_es | PDener ON PDsad&1; !Random slope for PDener_t regressed on PDsad_t-1 (cross-lag from sad to energetic)

beta_ea | PDener ON PDanxi&1; !Random slope for PDener_t regressed on PDanxi_t-1 (cross-lag from anxious to energetic)

beta_ev | PDener ON PDactv&1; !Random slope for PDener_t regressed on PDactv_t-1 (cross-lag from active to energetic)

logpi_s | PDsad; !random unique innovation variance for sad

logpi_a | PDanxi; !random unique innovation variance for anxious

logpi_v | PDactv; !random unique innovation variance for active

logpi_e | PDener; !random unique innovation variance for energetic

%BETWEEN%

!allow all 20 random effects to covary

phi_ss-logpi_e PDsad PDanxi PDactv PDener WITH

phi_ss-logpi_e PDsad PDanxi PDactv PDener;

Output: TECH1 TECH8 STDYX STAND(CLUSTER) FSCOMPARISON;

PLOT: TYPE = PLOT3; FACTOR =ALL;
